# The alterations in brain network functional gradients and dynamic functional connectivity in Alzheimer’s disease: a resting-state fMRI study

**DOI:** 10.3389/fnagi.2025.1716076

**Published:** 2026-01-07

**Authors:** Benqin Liu, Chunbing Chen, Pan Cai, Jiaren Zhang, Li Yang, Xu Chen, Xuejin Ma, Xuetao Jiang, Anjie Zhang, Linfeng Song, Lin Jiang

**Affiliations:** 1 Department of Radiology, The Third Affiliated Hospital of Zunyi Medical University (The First People’s Hospital of Zunyi), Zunyi, China; 2 Department of Traditional Chinese Medicine, The Third Affiliated Hospital of Zunyi Medical University (The First People’s Hospital of Zunyi), Zunyi, China

**Keywords:** Alzheimer’s disease, brain network, dynamic functional connectivity, functional gradient, resting-state functional magnetic resonance imaging

## Abstract

**Background and purpose:**

Alzheimer’s disease (AD), the most common form of dementia worldwide, is characterized by progressive cognitive decline. Extensive evidence from dynamic functional connectivity (dFC) studies has demonstrated unstable functional states, reduced network flexibility, and impaired transitions between large-scale neurocognitive networks across the AD continuum. However, how these temporal abnormalities are embedded within the hierarchical spatial organization of brain networks, as captured by functional gradients (FG), and whether combined FG-dFC metrics can provide mechanistically interpretable and potentially sensitive imaging biomarkers, remain to be elucidated.

**Methods:**

This study enrolled 46 AD patients who were diagnosed according to the Amyloid/Tau/Neurodegeneration (ATN) biological diagnostic framework and 37 age- and sex-matched healthy controls (HC). All participants underwent resting-state fMRI. Functional gradients were derived using connectivity similarity matrices and diffusion embedding (aligned and standardized), while dFC was estimated with a sliding window approach and clustered into four recurrent states. Group differences were assessed with two-sample *t*-tests with Gaussian Random Field (GRF) correction. Correlation analyses included ATN biomarkers and cognitive scores. A linear support vector machine (SVM) with leave-one-out cross-validation evaluated classification performance based on significant FG features.

**Results:**

Compared to the healthy controls, AD patients exhibited widespread FG alterations between regions of the Default Mode Network (DMN) and the Sensorimotor Network (SMN). In the first gradient DMN, the left precuneus showed reduced gradient scores, whereas the right medial superior frontal gyrus and bilateral angular gyri were increased. In the first gradient of the SMN, the right supplementary motor area increased while bilateral superior temporal gyri decreased. Second-gradient reductions were confined to two regions: the left postcentral gyrus (SMN) and left middle occipital gyrus (visual network, VIS). The right medial superior frontal gyrus first-gradient score correlated negatively with T-Tau (*r* = −0.50, *P* = 0.006) and age (*r* = −0.36, *P* = 0.02); the right angular gyrus correlated negatively with age (*r* = −0.29, *P* = 0.04); the left precuneus correlated positively with age (*r* = 0.38, *P* = 0.009). dFC revealed four recurrent states (27.59, 17.67, 28.27, 26.47% of total occurrences). Relative to HC, AD showed higher FT and MDT in states 1–2 and lower scores in state 3, with NT unchanged, alongside state-dependent bidirectional connectivity changes (fronto-insular-sensorimotor increases; DMN-temporal and visuo-auditory decreases). The SVM achieved an AUC of 0.776, sensitivity 78.26%, specificity 67.57%, and accuracy 73.49%, with the right superior temporal gyrus within SMN first-gradient contributing most.

**Conclusion:**

AD is characterized by macro-scale hierarchical disorganization centered on the principal functional gradient, accompanied by reduced cross-state flexibility and state-dependent connectivity abnormalities. The combined functional gradient-dynamic functional connectivity (FG-dFC) analysis provides complementary spatiotemporal insights and reveals imaging features associated with T-Tau levels and age, offering new perspectives on the neuropathological mechanisms of AD and potential imaging biomarkers. Moreover, these network topology and dynamic connectivity metrics may prove useful for monitoring disease progression, evaluating treatment effects, and stratifying patients in future clinical and interventional studies.

## 1 Introduction

In recent years, resting-state functional magnetic resonance imaging (rs-fMRI), a safe and non-invasive imaging technique, has been widely applied to the early diagnosis and mechanistic study of AD (Wang et al., 2015). Conventional rs-fMRI studies have focused on alterations in static functional connectivity (sFC), which evaluates network integration and segregation by calculating the average correlation between brain regions across the entire scanning period (Trieu et al., 2024). Prior studies have reported characteristic abnormalities in AD, including reduced intra-DMN connectivity and decoupling between the DMN and other large-scale networks (Sintini et al., 2024). However, since sFC assumes stationarity, it fails to account for the time-varying nature of brain connectivity, thereby limiting its ability to capture crucial physiological features such as network fluctuations, abrupt transitions, and compensatory mechanisms.

To overcome this limitation, dynamic functional connectivity (dFC) approaches have been increasingly applied in AD research. By modeling time-resolved connectivity patterns and recurrent brain states using sliding-window or related methods (Long et al., 2023), a growing body of literature has demonstrated that AD is characterized by destabilized functional states, reduced flexibility, and impaired transitions between large-scale neurocognitive networks across the disease continuum, from preclinical stages to overt dementia (Canal-Garcia et al., 2024). These alterations have been reported in both seed-based and whole-brain network analyses and have been shown to relate closely to cognitive decline, molecular pathology, and disease severity (Pini et al., 2025b; Tessadori et al., 2025). Collectively, these studies highlight that temporal properties of large-scale brain networks are highly sensitive to AD-related pathology and capture aspects of network dysfunction that are not observable using conventional sFC measures.

In parallel, the functional gradient (FG) approach has provided a new pathway for untangling the spatial organizational principles of large-scale brain networks (Margulies et al., 2016). Using dimensionality reduction techniques to extract the principal components of connectivity similarity between regions, FG map continuous functional axes spanning from primary sensorimotor cortices to higher-order transmodal association areas (such as DMN hubs), thereby revealing hierarchical pathways of information processing in the brain (Jiang et al., 2023). Existing studies have indicated that AD patients exhibit blurred FG, cortical dedifferentiation, and reduced resolution of network segregation, suggesting a regression of the neural system from a structured, specialized state toward a more diffuse, non-specific organization (Ottoy et al., 2024; Wang D. et al., 2024). Despite the extensive literature on dFC alterations and the emerging evidence for FG reorganization in AD, it remains largely unknown how temporal disruptions in network dynamics are embedded within the brain’s hierarchical spatial architecture. Most existing dFC studies focus on macroscopic temporal metrics-such as connectivity strength, state dwell time, or transition frequency-without explicitly considering their spatial positioning along intrinsic functional gradients. Consequently, an integrative framework that links temporal network dynamics to hierarchical spatial organization is still lacking in AD research.

Therefore, the present study integrates FG and dFC analyses to jointly evaluate the dynamic variability and hierarchical organization of multiple brain networks in AD. We further employ machine learning methods to identify and validate key gradient-based features, explore their potential as neuroimaging biomarkers, and investigate their neurobiological correlates using cognitive assessments and fluid biomarkers (e.g., T-Tau). By adopting a dual-dimensional perspective of “temporal fluctuation-spatial topology,” this study aims to provide more comprehensive evidence for understanding the mechanisms of brain network degeneration and the neural basis of cognitive impairment in AD.

## 2 Materials and methods

### 2.1 Participants

A total of 46 patients with AD were recruited from the Department of Cerebrovascular Diseases at the Third Affiliated Hospital of Zunyi Medical University (the First People’s Hospital of Zunyi City). Additionally, 37 healthy controls (HC) matched for age, sex, education level, and other relevant factors were enrolled. The recruitment of AD patients was based on the 2018 ATN biological diagnostic framework jointly established by the National Institute on Aging and the Alzheimer’s Association (NIA-AA). Enrolled AD patients were required to meet the criteria for A + (amyloid-β positive) along with either T+ or N+ , supporting the biological definition of typical AD (Jack et al., 2018). Ethics number: 2025-1-736.

### 2.2 Inclusion and exclusion criteria

This study enrolled patients with AD (*n* = 46; 22 males, 24 females) with a mean age of 68.07 ± 6.46 years. The inclusion criteria were as follows: (1) AD diagnosis was based on the 2018 National Institute on Aging-Alzheimer’s Association (NIA-AA) ATN biological classification framework, meeting the biological definition of AD with any of the following biomarker profiles (Jack et al., 2018): ➀ A + T + (positive for amyloid-β based on cerebrospinal fluid (CSF) or positron emission tomography (PET), along with positive phosphorylated Tau protein), or ➁ A + N + (positive for amyloid-β accompanied by evidence of neurodegeneration, such as MRI atrophy, hypometabolism on FDG-PET, or elevated CSF total Tau); (2) age between 55 and 80 years; and (3) ability to complete MRI scanning, neuropsychological assessments, and collection of fluid biomarkers. Exclusion criteria included: (1) absence of amyloid-β deposition (A−); (2) meeting clinical diagnostic criteria for other neurodegenerative diseases [e.g., frontotemporal dementia or Dementia with Lewy Bodies (DLB)]; (3) comorbid major psychiatric disorders or metabolic diseases contributing to cognitive impairment; and (4) inability to complete MRI, lumbar puncture, or neuropsychological evaluations. On the same day as the MRI scan, all AD patients underwent assessments using the Mini-Mental State Examination (MMSE), Montreal Cognitive Assessment (MoCA), and Clinical Dementia Rating (CDR). Additionally, HC participants (*n* = 37; 18 males, 19 females) with a mean age of 65.43 ± 8.40 years were enrolled. Inclusion criteria for the HC group were: (1) biomarker profile consistent with the “A−T−N−” category per the ATN framework, specifically: ➀ no Aβ deposition (A−): normal CSF Aβ42 levels; ➁ absence of Tau pathology (T−): normal phosphorylated Tau (p-Tau); and ➂ no neurodegeneration (N−): no MRI atrophy or normal CSF total Tau levels; (2) age between 55 and 80 years, matching the AD group; and (3) no significant structural abnormalities on MRI, such as atrophy, infarction, or hemorrhage. Exclusion criteria for the HC group were: (1) positivity for any ATN biomarker (A + , T + , or N +); (2) history of any neurological or psychiatric disorders; and (3) poor image quality.

### 2.3 MRI data acquisition

MRI data were acquired on a Siemens MAGNETOM Vida 3.0 T MRI system equipped with a 20-channel head coil. Conventional MRI sequences, including axial T_1_-weighted imaging (T_1_WI), axial T_2_-weighted imaging (T_2_WI), and T_2_-fluid attenuated inversion recovery (T_2–_FLAIR), were first acquired to exclude participants with macroscopic structural brain abnormalities. Subsequently, 3D T_1_-weighted images were obtained using a magnetization-prepared rapid acquisition gradient echo (MPRAGE) sequence, followed by blood oxygen level-dependent (BOLD) imaging. During scanning, all participants were placed in a supine position, instructed to remain still with their eyes closed. Head motion was minimized using foam padding, and soft earplugs were provided to reduce noise interference.

3D-T_1_WI-MPRAGE: repetition time (TR) = 1,650.0 ms, echo time (TE) = 2.43 ms, voxel size = 0.9 × 0.9 × 1 mm, flip angle = 8°, field of view (FOV) = 230 mm × 230 mm, slice thickness = 1 mm, and number of pulses = 1. For the BOLD sequence, the parameters were: repetition time (TR) = 2,000.0 ms, echo time (TE) = 30.0 ms, voxel size = 2.9 × 2.9 × 3.0 mm, field of view (FOV) = 240 mm × 240 mm, number of slices = 50, and slice thickness = 3.0 mm.

The preprocessing of 3D-T_1_WI-MPRAGE and BOLD images was performed using SPM,^1^ DPARSF,^2^ and custom code in MATLAB (The MathWorks, Natick, MA, United States). Initially, the raw DICOM data were converted to the NIFTI format. The first 10 functional volumes were discarded to allow for signal equilibration and environmental adaptation. The remaining 230 images were corrected for slice-timing differences using sinc interpolation and subsequently realigned to the first volume for head motion correction. Subjects exhibiting head motion exceeding 2 mm in any of the x, y, or z directions and/or a rotation angle greater than 2° were excluded. The BOLD functional images were co-registered with high-resolution T_1_-weighted images and normalized to the Montreal Neurological Institute (MNI) space (resampled voxel size = 3 mm × 3 mm × 3 mm). Noise covariates, such as cerebrospinal fluid signals, were regressed out, and spatial smoothing was applied using a 6 mm full-width at half-maximum (FWHM) Gaussian kernel. The selection of kernel size influences subsequent analyses: larger kernels (e.g., 8 mm or 10 mm FWHM) enhance statistical sensitivity at the expense of spatial specificity, whereas smaller kernels (e.g., 4 mm FWHM) preserve finer anatomical details but may constrain the detection of network-level effects. A 6 mm FWHM kernel was chosen to balance spatial resolution and noise reduction, consistent with common practice in resting-state fMRI studies (Candemir, 2023). Additionally, detrending was performed to remove linear and non-linear trends from the time-series data. Finally, temporal band-pass filtering was applied within the frequency range of 0.01–0.08 Hz.

### 2.4 Dynamic functional connectivity analysis

To construct FG of brain networks, we extracted the BOLD time series from the seven brain networks defined by the Yeo atlas (Bayrak et al., 2019) (visual, somatomotor, dorsal attention, ventral attention, limbic, frontoparietal, and default mode networks). Additionally, the cerebral cortex was parcellated into 400 regions, and the mean BOLD time series of each region was extracted. For each voxel within each brain network, the Pearson correlation coefficient was computed between the BOLD time series of the voxel and the mean time series of each cortical region, resulting in a functional connectivity matrix for each network per subject. These functional connectivity matrices were normalized using Fisher’s *r*-to-*z* transformation. Following previous studies, the functional connectivity matrices were used to compute cosine similarity matrices, representing the connectivity similarity between each pair of voxels within each network for each subject. This process generated a similarity matrix for each participant to capture spatial microstructure patterns. Using the Brain Space toolbox,^3^ the gradients of the seven brain networks were computed. Diffusion map embedding was implemented using the normalized graph-Laplacian algorithm in the BrainSpace toolbox. First, a voxelwise cosine similarity matrix was transformed into a symmetric affinity matrix and normalized to yield a Markov transition matrix (Bi et al., 2018), which describes the probabilistic diffusion of connectivity patterns across the cortical manifold. The eigen decomposition of this matrix produced a set of orthogonal eigenvectors (gradients) ranked by descending eigenvalues, each representing a continuous axis of functional variation. The first principal gradient typically reflects a unimodal-to-transmodal transition from primary sensorimotor cortices to higher-order association areas, while subsequent gradients capture secondary modes of functional differentiation. Parameter α was set to 0.5 to balance the influence of local and global relationships, and diffusion time *t* was fixed at 0 to avoid iterative smoothing, consistent with previous gradient-mapping studies (Thomas Yeo et al., 2011; Hong et al., 2019). This approach preserves the intrinsic manifold geometry of the high-dimensional functional connectivity space and allows robust comparison of gradient topology across participants. A group-level gradient template was generated based on the average functional connectivity matrix across all participants. Subsequently, Procrustes alignment was applied to estimate individual gradients and align them to the group template. These aligned gradients were then smoothed and standardized into z-scores for further analysis. We focused on the principal gradient component, which accounted for the largest proportion of variance and exhibited the highest interpretability. To capture the spatial distribution of each network within the functional gradient space, we calculated the gradient dispersion for each participant by measuring the Euclidean distance between the centroids of regions belonging to each network in the aligned gradient space. This gradient space was defined by the dominant FG of each network, allowing us to quantify the extent to which brain network regions were functionally integrated with or segregated from others based on Euclidean distance calculations (Coifman et al., 2005; Thomas Yeo et al., 2011).

### 2.5 Discriminative efficacy of support vector machine in predicting functional gradient features

A linear support vector machine (SVM) was employed as the primary supervised learning classifier to evaluate the significance of FG features in patients with AD. Brain regions exhibiting significant differences in FG within the seven major networks between AD patients and HC were used as predictive features. Prediction accuracy was estimated using leave-one-out cross-validation (LOOCV). The principle of LOOCV is to iteratively leave out one sample from the dataset for testing while using all remaining samples for training. In other words, for a dataset containing N samples, LOOCV performs N rounds of training and testing, each time using N-1 samples as the development set and one sample as the test set. The performance of the proposed machine learning method was evaluated by compiling true positive, true negative, false positive, and false negative outcomes into a confusion matrix. Subsequently, metrics including accuracy, precision, sensitivity, specificity, area under the curve (AUC), and receiver operating characteristic (ROC) scores were calculated for the machine learning model.

### 2.6 Dynamic functional connectivity analysis

dFC maps for each participant were obtained using the sliding window approach via the DynamicBC toolbox.^4^ In sliding window-based dFC analysis, window length is a critical parameter. In this study, the window length was set to 50 TRs with a step size of 1, resulting in a total of 90 windows for investigating dynamic functional connectivity (Christiaen et al., 2020; Li et al., 2024). This window length (∼100 s) provides an optimal balance between temporal resolution and estimation reliability. Shorter windows (< 30 TRs) can increase sensitivity to transient fluctuations but often yield unstable correlation estimates due to limited samples, whereas longer windows (> 80 TRs) may excessively smooth temporal variations and obscure short-lived connectivity states (Shakil et al., 2016; Liuzzi et al., 2019). The 50-TR window length has been validated in both simulation and empirical AD studies as suitable for detecting network state transitions within low-frequency (< 0.1 Hz) BOLD fluctuations (Schumacher et al., 2019). The step size of 1 TR ensures fine-grained temporal continuity and minimizes redundancy between adjacent windows, enhancing the robustness of dynamic state identification (Sahib et al., 2018; Savva et al., 2019). Within each sliding time window, correlation coefficients were computed between the time series of each cortical region and all other voxels, generating a series of sliding-window correlation maps for each participant. To improve the normality of the correlation distribution, Fisher’s *z*-transformation was applied to the correlation maps. To assess dFC variability, the standard deviation of the *z*-scores was calculated for each voxel, representing the variance of the time series and correlation coefficients. The K-means clustering algorithm was then employed to perform clustering analysis on the functional connectivity matrices of each sliding window. The K-means algorithm involves initializing cluster centers, assigning each data point to the nearest center, updating the centers, and iterating this process until cluster centers stabilize (Saha et al., 2021). The number of clusters (*k*) was optimized using the elbow method to determine the optimal value. To identify the best *k*, the sum of squared errors (SSE) was examined for *k* scores ranging from 2 to 20, and the SSE curve was plotted. The analysis revealed a matrices of each *k* = 4, indicating a balance between model complexity and variance explained. Furthermore, the Silhouette coefficient and the Calinski-Harabasz index were calculated to evaluate clustering stability. Both metrics indicated that *k* = 4 provided the most robust separation between states while maintaining within-cluster cohesion. Therefore, *k* = 4 was selected. Finally, the K-means clustering analysis revealed dynamic network patterns of the brain across different functional states, and differences in functional connectivity between the AD group and the HC group were compared 0.05.

### 2.7 Statistical analysis

Two-sample *t*-tests were employed to compare functional gradient scores between the AD and HC groups, with age and sex included as covariates. The statistical significance threshold was set at *P* < 0.05. Multiple comparisons were corrected using the false discovery rate (FDR) method at the network level and the GRF correction at the voxel level (*P* < 0.05, cluster-level corrected). Pearson or Spearman correlation coefficients were calculated between abnormal gradient scores in the AD group and variables including age, sex, years of education, Aβ42, Aβ40, Aβ42/Aβ40 ratio, T-Tau, P-Tau, MMSE, MoCA, and CDR scores. The significance level was set at *P* < 0.05.

## 3 Results

### 3.1 Demographic and clinical data

A total of 50 participants were initially recruited into the AD group, of whom 46 were finally included (22 males and 24 females, aged 56–78 years). Forty HC participants were recruited, and 37 were ultimately included after excluding those with excessive head motion (18 males and 19 females, aged 55–84 years). No statistically significant differences were observed in age or gender between the AD and HC groups (*P* > 0.05) (Table 1).

**TABLE 1:** Demographic characteristics of the sample.

Characteristics	AD	HC	*t*/χ^2^	*P*
Number of subjects	46	37	–	–
Sex (male/female)	22/24	18/19	0.006^a^	0.941
Age (years)	68.07 ± 6.46	65.43 ± 8.40	0.110^b^	1.615
MMSE	9 (1.5, 13)	–	–	–
MOCA	7.50 (0, 12)	–	–	–
CDR	3 (2, 3)	–	–	–
Aβ42	514.70 ± 185.20	–	–	–
Aβ40	21,121 ± 6,361	–	–	–
T-Tau	707.90 ± 373.70	–	–	–
P-Tau	98.82 (59.36, 138.10)	–	–	–

AD, Alzheimer’s disease; HC, healthy controls; MMSE, Mini-Mental State Examination; MoCA, Montreal Cognitive Assessment; CDR, Clinical Dementia Rating.

^a^Indicates χ^2^ value;

^b^indicates *t*-value.

### 3.2 FG

#### 3.2.1 FG of the DMN, SMN, and VIS

**FIGURE 1:**
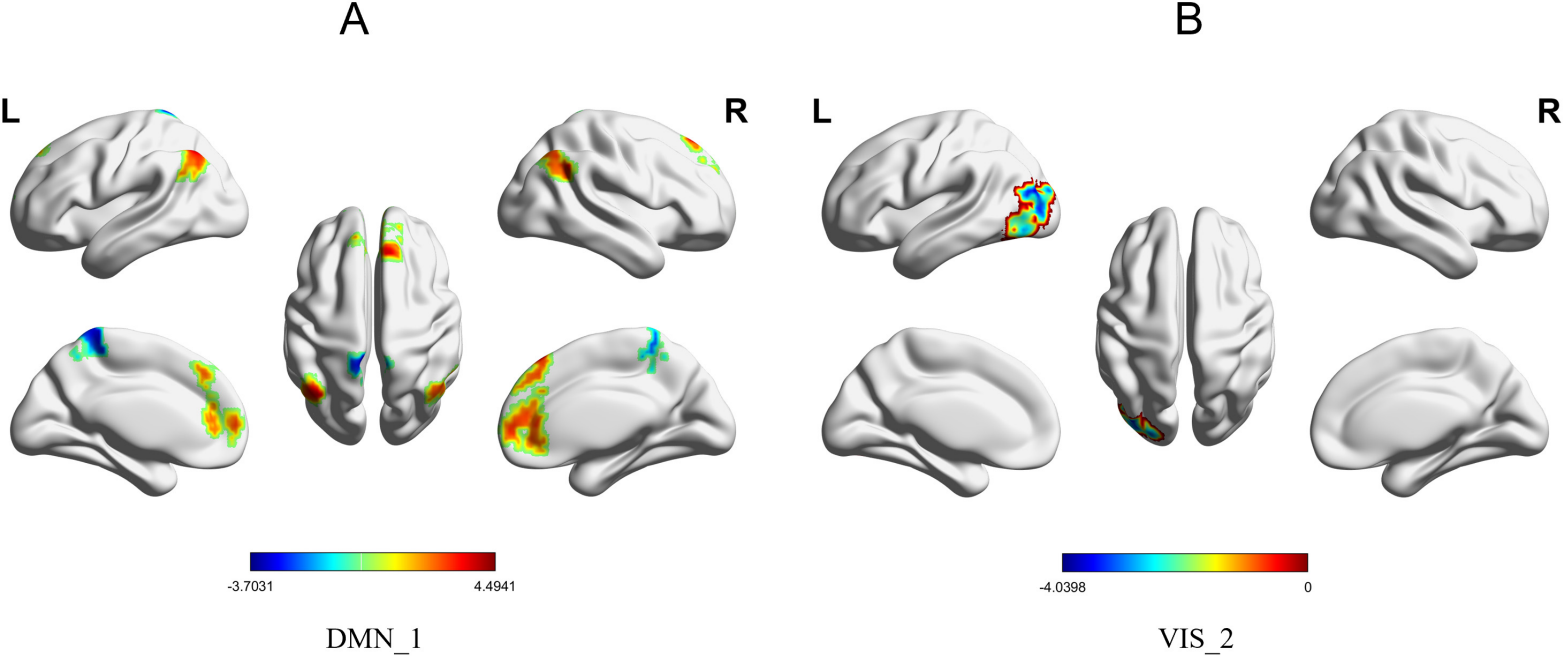
Brain regions with significant functional gradient differences in the DMN/VIS. **(A)** Significant between-group differences in Gradient 1 between the AD group and the HC group were observed in the right medial superior frontal gyrus, right angular gyrus, left precuneus, and left angular gyrus within the DMN. The surface rendering was generated using BrainNet Viewer. **(B)** Significant between-group differences in Gradient 2 of the VIS were observed in the left middle occipital gyrus.

**FIGURE 2:**
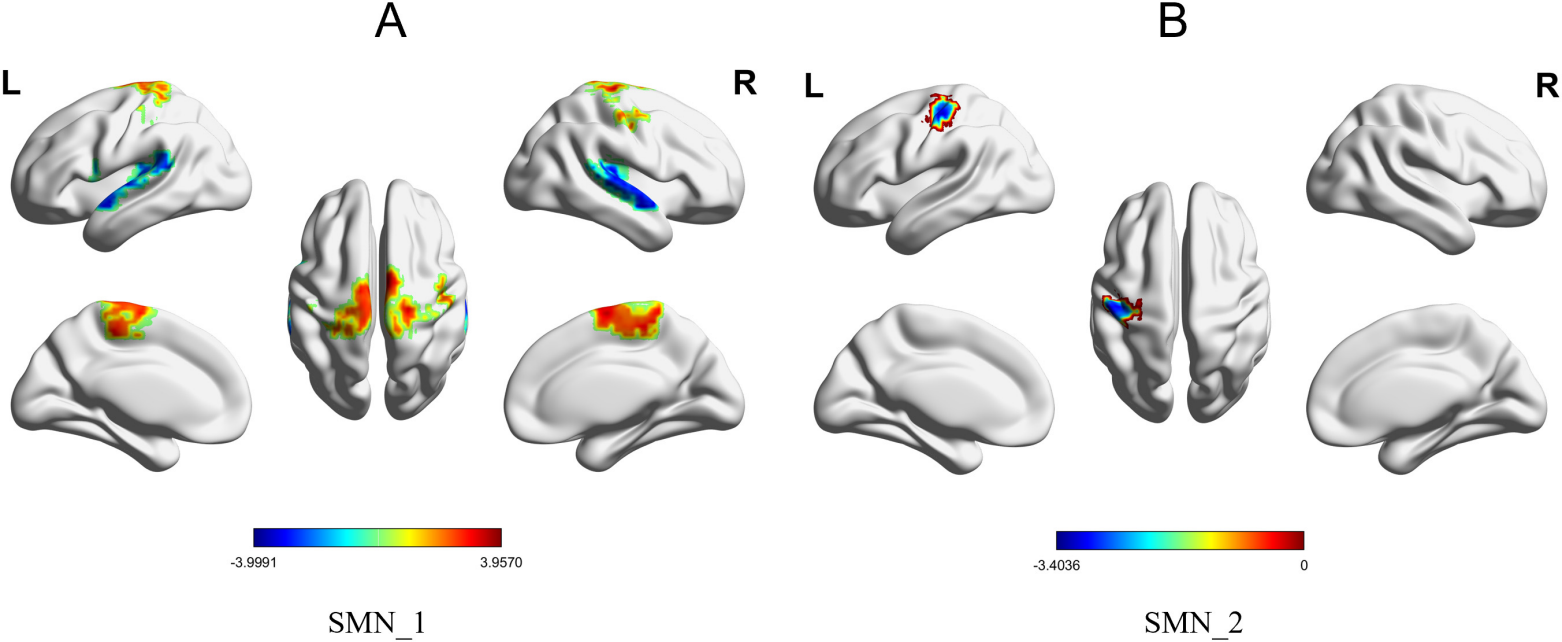
Brain regions with significant functional gradient differences in the SMN. **(A)** Significant between-group differences in Gradient 1 between the AD group and the HC group were observed in the right supplementary motor area, right superior temporal gyrus, and left superior temporal gyrus within the SMN. The surface rendering was generated using BrainNet Viewer. **(B)** A significant between-group difference in Gradient 2 between the two groups was observed in the left postcentral gyrus within the SMN.

The brain regions within the DMN, SMN, and VIS that exhibited significant between-group differences in FG among all AD patients included in this study are presented in Table 2. We analyzed the first to the third principal gradient components of the seven brain networks in AD patients and identified significant between-group differences in the DMN, SMN, and VIS. The results revealed four brain regions with significant between-group differences in the first gradient component of the DMN: the functional gradient scores of the left precuneus was lower in the AD group than in the HC group, whereas the scores in the right superior medial frontal gyrus, right angular gyrus, and left angular gyrus were higher in the AD group than in the HC group (Figure 1A). For the SMN, three brain regions showed significant between-group differences in the first gradient component: the functional gradient scores of the right supplementary motor area was higher in the AD group than in the HC group, while the scores in the right superior temporal gyrus and left superior temporal gyrus were lower in the AD group than in the HC group (Figure 2A). Additionally, one brain region-the left postcentral gyrus-exhibited a significant between-group difference in the second gradient component, with a lower functional gradient value in the AD group compared to the HC group (Figure 2B). In the VIS, only the left middle occipital gyrus in the second gradient component showed a significant between-group difference, with a lower functional gradient value in the AD group compared to the HC group (Figure 1B). All between-group differences were assessed using two-tailed *t*-tests with (*P* < 0.05), corrected by Gaussian random field (GRF) method.

**TABLE 2:** Brain regions with significant differences in DMN/SMN/FPN gradient scores between the AD and HC groups.

Network (Gradient)	Brain regions (AAL3)	Voxel*s*, *n*	MNI coordinates, mm (x, y, z)			*t*
**DMN**						
Gradient 1	Frontal_Sup_Medial_R	765	12	36	54	4.1769
Gradient 1	Angular_R	292	60	−54	36	3.7399
Gradient 1	Precuneus_L	268	−3	−42	66	−3.7031
Gradient 1	Angular_L	218	−39	−66	−48	3.9384
**SMN**						
Gradient 1	Supp_Motor_Area_R	1,155	9	−6	66	3.9570
Gradient 1	Temporal_Sup_R	567	36	−30	12	−3.9991
Gradient 1	Temporal_Sup_L	409	−57	0	−12	−3.8107
Gradient 2	Postcentral_L	216	−51	−21	63	−3.4036
**VIS**						
Gradient 2	Occipital_Mid_L	456	−33	−84	3	−4.0399

GRF, Gaussian Random Field correction (voxel *P* < 0.05, cluster *P* < 0.05); MNI, Montreal Neurological Institute; AAL, Automated Anatomical Labeling; GRF, Gaussian Random Field; DMN, Default Mode Network; SMN, Sensorimotor Network; VIS, Visual Network.

#### 3.2.2 Discriminative efficacy of support vector machine in predicting functional gradient features

**FIGURE 3:**
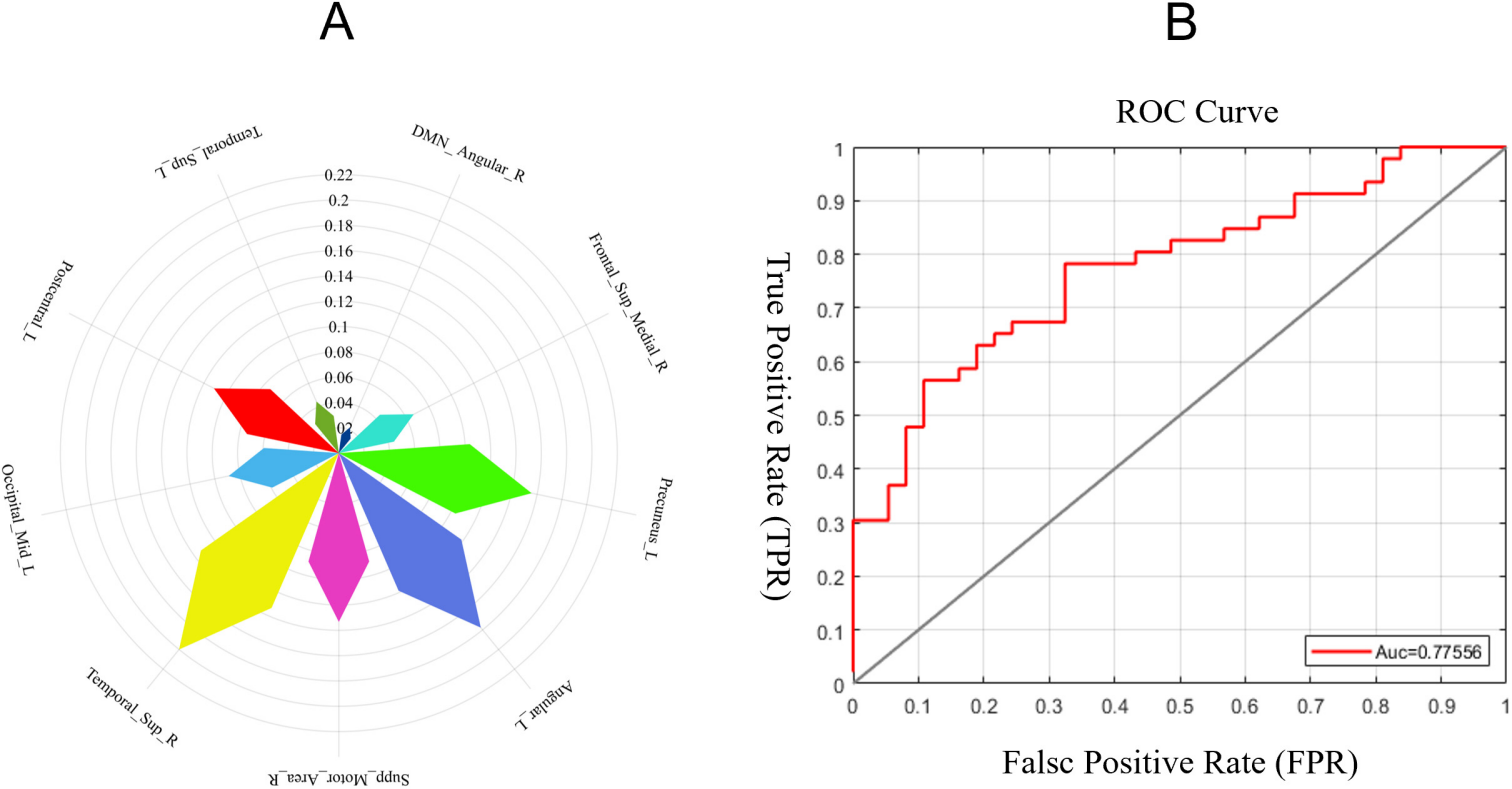
Radar and ROC curves. **(A)** Network-level comparisons of the first three gradients between the AD group and the HC group revealed significant differences. The radar plot highlights the discriminative efficacy of each region. The most discriminative region was the right superior temporal gyrus within the first gradient of the SMN. **(B)** The AUC-ROC plot derived from the support vector machine (SVM) importance ranking shows an AUC value of 73.49%.

To evaluate the discriminative efficacy of functional gradient features derived from brain regions showing significant intergroup differences between AD and HC groups in classifying AD patients, a support vector machine (SVM) model was employed. The results indicated that the first principal component of the functional gradient in the right superior temporal gyrus within the SMN exhibited high discriminative power in this study (Figure 3A). The receiver operating characteristic (ROC) curve is shown in Figure 3B. The area under the curve (AUC), sensitivity, specificity, and accuracy are presented in Table 3.

**TABLE 3:** Performance metrics of SVM.

AUC	Sensitivity (%)	Specificity (%)	Accuracy (%)
7.56	78.26	67.57	73.49

AUC, area under the curve.

### 3.3 Dynamic functional connectivity

#### 3.3.1 dFC clustering analysis and functional connectivity in dynamic states

**FIGURE 4:**
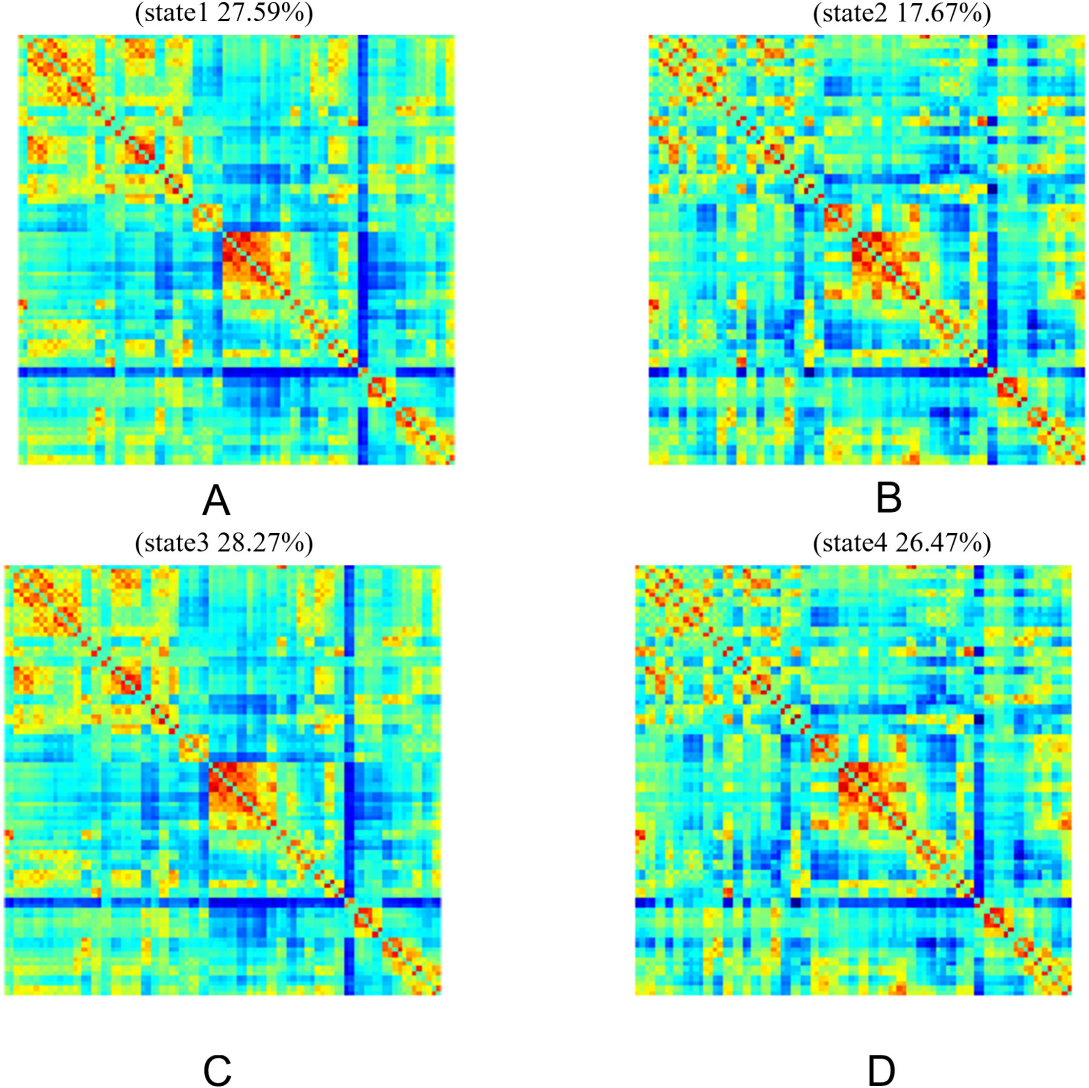
Brain feature clustering in State 1, State 2, State 3, and State 4. **(A-D)** Brain feature clustering at *k* = 4, showing the cluster centroids for State 1, State 2, State 3, and State 4, along with the percentage occurrence of each state.

Using the k-means clustering method, four highly structured functional connectivity states were identified, which recurred across individual scans and among different subjects. The total occurrence proportions of these four states varied across all subjects (Figures 4A–D). Specifically, State 1 accounted for 27.59%, State 2 for 17.67%, State 3 for 28.27%, and State 4 for 26.47%.

**FIGURE 5:**
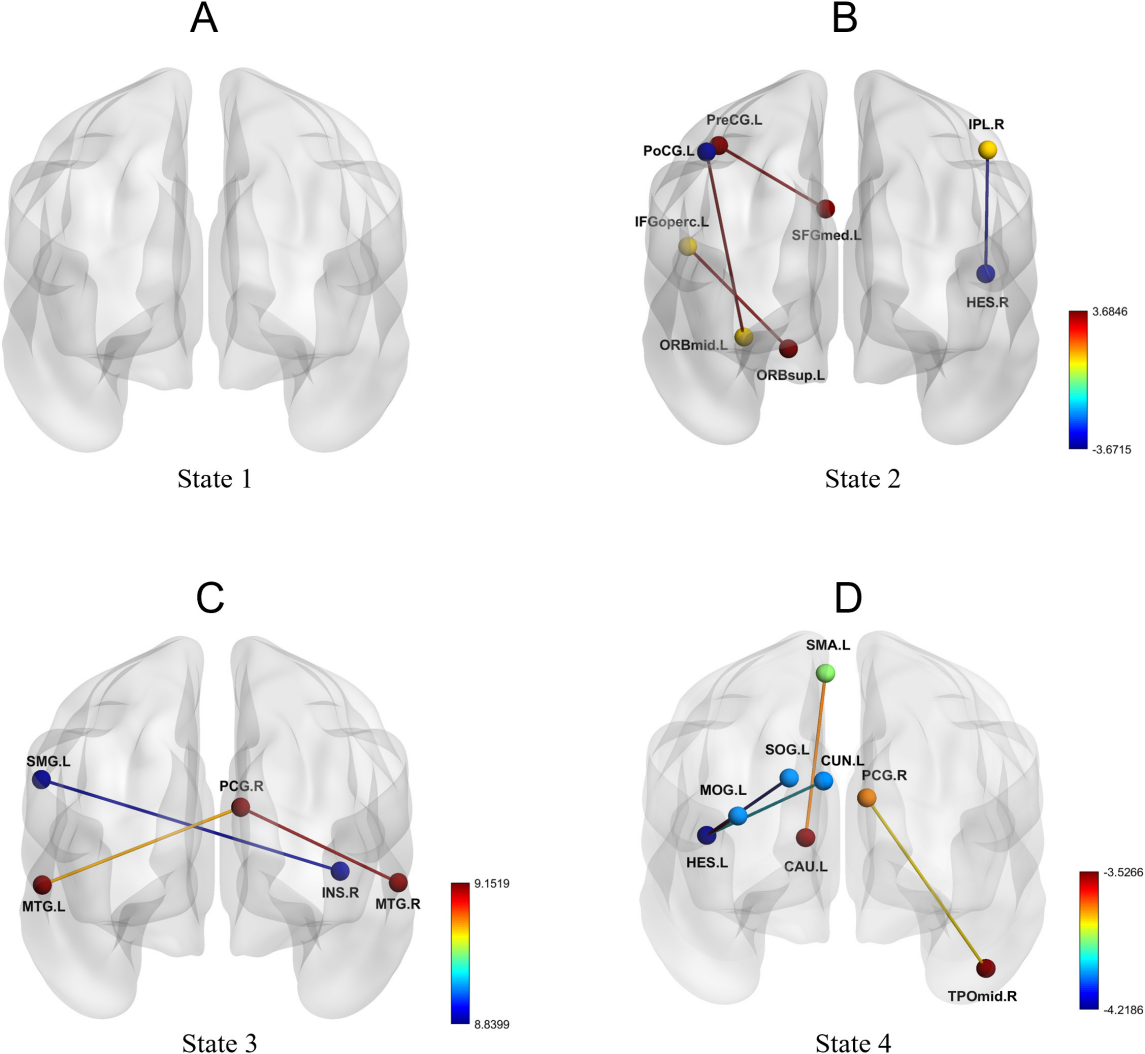
Dynamic functional connectivity patterns in State 1, State 2, State 3, and State 4. **(A-D)** Changes in dynamic functional connectivity differences between the AD group and the HC group within State 1, State 2, State 3, and State 4.

Furthermore, dFC analysis based on the sliding window approach revealed increased connectivity between multiple brain regions in the AD group. Figure 5 illustrates the functional connectivity of various brain regions in each state. No significant changes in connectivity were observed in State 1 (Figure 5A). In State 2, enhanced connectivity was detected between the left orbital part of the superior frontal gyrus and the left opercular part of the inferior frontal gyrus, between the left precentral gyrus and the left medial superior frontal gyrus, and between the left orbital part of the middle frontal gyrus and the left postcentral gyrus. Conversely, weakened connectivity was observed between the right angular gyrus and the right transverse temporal gyrus (Table 4; Figure 5B). In State 3, reduced connectivity was identified between the right insula and the left supramarginal gyrus, between the right posterior cingulate gyrus and the left middle temporal gyrus, and between the right posterior cingulate gyrus and the right middle temporal gyrus (Table 5; Figure 5C). In State 4, decreased connectivity was noted between the left supplementary motor area and the left caudate nucleus, between the left cuneus and the left transverse temporal gyrus, between the left superior occipital gyrus and the left transverse temporal gyrus, between the left middle occipital gyrus and the left transverse temporal gyrus, and between the right posterior cingulate gyrus and the right middle temporal gyrus (Table 6; Figure 5D).

**TABLE 4:** Dynamic functional connectivity differences in state 2.

Brain regions	*t*	*p-FDR*
ORBsup.L—IFGoperc.L	3.582	<0.001
PreCG.L—SFGmed.L	3.685	<0.001
ORBmid.L—PoCG.L	3.648	<0.001
IPL.R—HES.R	−3.672	<0.001

**TABLE 5:** Dynamic functional connectivity differences in state 3.

Brain regions	*t*	*p-FDR*
INS.R—SMG.L	−8.840	<0.0001
PCG.R—MTG.L	−9.058	<0.0001
PCG.R—MTG.R	−9.152	<0.0001

**TABLE 6:** Dynamic functional connectivity differences in state 4.

Brain regions	*t*	*p-FDR*
SMA.L—CAU.L	−3.706	<0.001
CUN.L—HES.L	−3.987	<0.001
SOG.L—HES.L	−4.219	<0.001
MOG.L—HES.L	−3.527	<0.001
PCG.R—TPOmid.R	−3.772	<0.001

**FIGURE 6:**
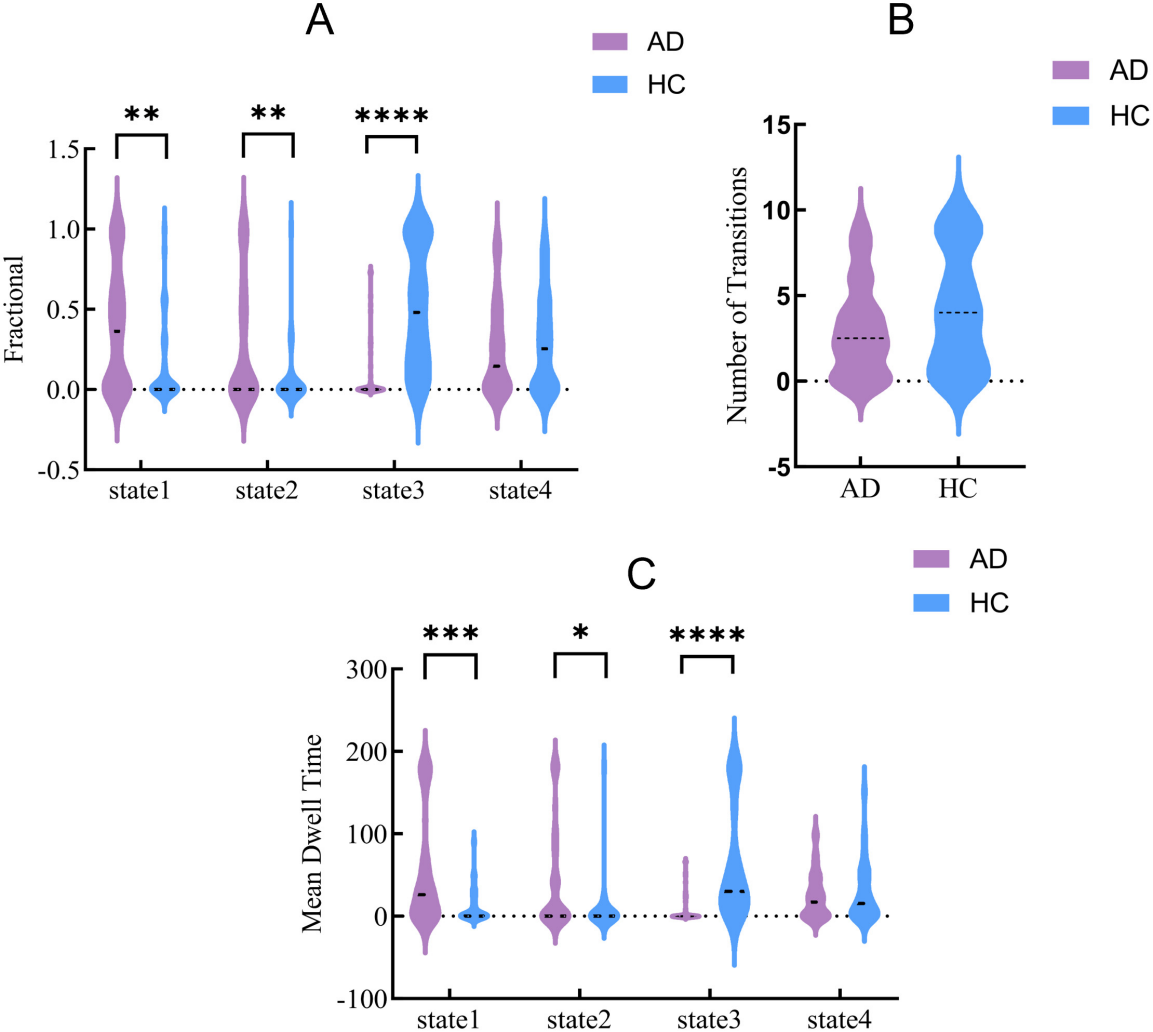
Between-group differences in temporal metrics across dynamic functional connectivity states. **(A)** Fractional occupancy (FO), **(B)** number of transitions (NT), and **(C)** mean dwell time (MDT) across four states in the AD and HC groups. Violin plots show the distributions for AD (purple) and HC (blue). The symbols indicate levels of statistical significance: *p < 0.05, **p < 0.01, ***p < 0.001, ****p < 0.0001. AD, Alzheimer’s disease; HC, healthy control.

Statistical comparisons between the AD group and the HC group in terms of fractional occupancy (FT), mean dwell time (MDT), and number of transitions (NT) revealed no significant difference in NT (Figure 6B). However, compared with the HC group, the AD group exhibited increased FT and MDT in State 1 and State 2 (*P* < 0.05), and decreased FT and MDT in State 3 (*P* < 0.05) (Figures 6A,C).

### 3.4 Correlation analysis between variables and clinical data

**FIGURE 7:**
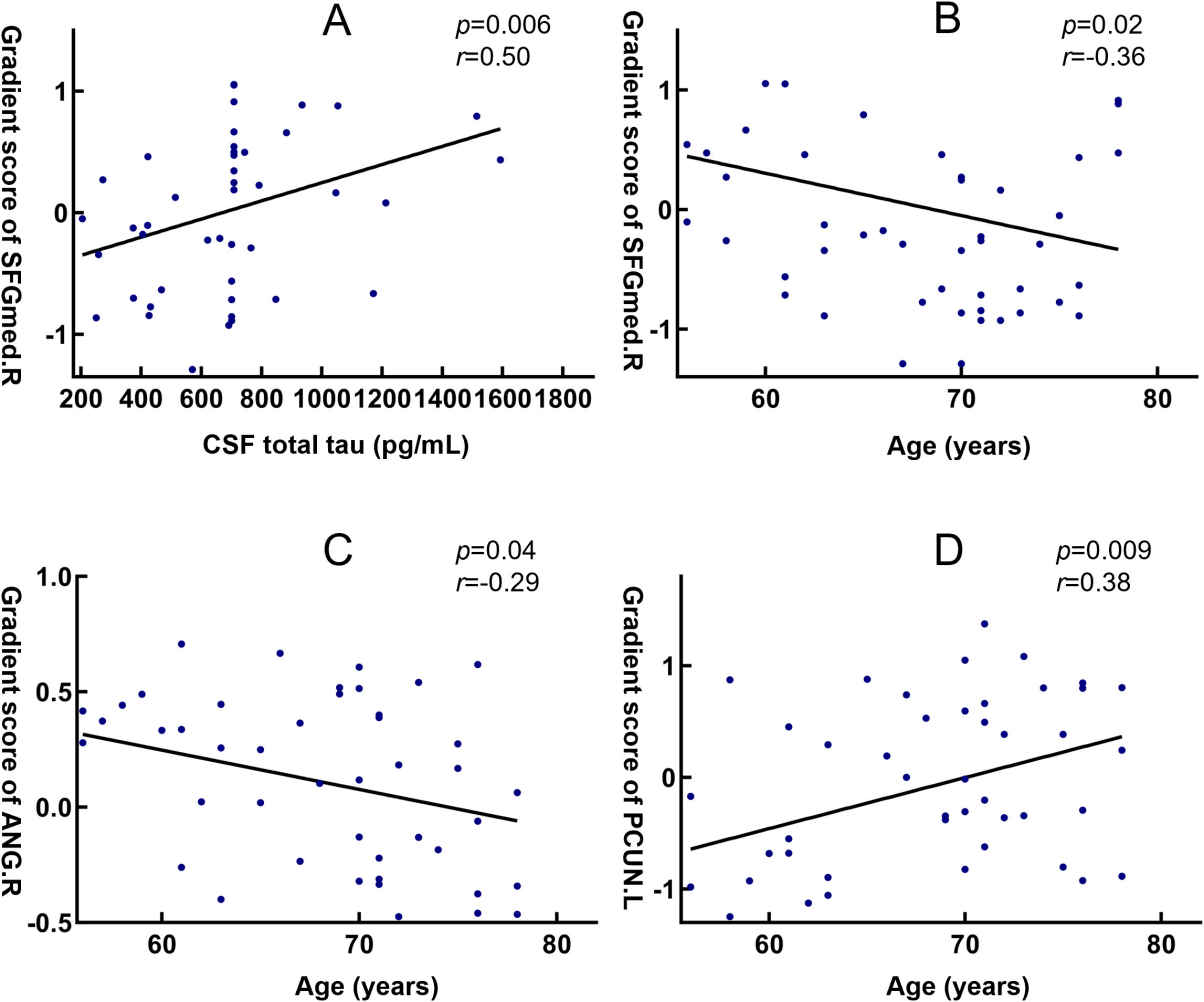
Correlation between brain regions with functional gradient differences and clinical variables. **(A)** Spearman correlation analysis shows the association between the gradient value of the right medial superior frontal gyrus and T-Tau in patients with AD; **(B)** Spearman correlation analysis shows the association between the gradient value of the right medial superior frontal gyrus and age in AD patients; **(C)** Spearman correlation analysis shows the association between the gradient value of the right angular gyrus and age in AD patients; **(D)** Spearman correlation analysis shows the association between the gradient value of the left precuneus and age in AD patients. FGmed.R, right medial superior frontal gyrus; ANG.R, right angular gyrus; PCUN.L, left precuneus.

In the analysis of the first gradient of the DMN in patients with AD, several brain regions exhibiting significant differences were found to correlate with clinical variables. Specifically, the gradient score of the right superior medial frontal gyrus showed a significant positive correlation with T-tau levels (*r* = −0.50, *P* = 0.006) and a significant negative correlation with age (*r* = −0.36, *P* = 0.02) (Figures 7A,B). The gradient score of the right angular gyrus was significantly negatively correlated with age (*r* = −0.29, *P* = 0.04) (Figure 7C). In contrast, the gradient value of the left precuneus demonstrated a significant positive correlation with age (*r* = 0.38, *P* = 0.009) (Figure 7D).

## 4 Discussion

Based on resting-state functional magnetic resonance imaging (rs-fMRI) and employing functional gradient and sliding window approaches, this study systematically untangled abnormalities in the hierarchical organization and dynamic connectivity features across multiple brain networks in patients with AD. The results revealed that functional gradient abnormalities in AD patients were primarily concentrated in the first gradient, with a minority observed in the second gradient, while no significant differences were detected in the third gradient. Furthermore, dFC analysis identified a bidirectional pattern of both enhanced and weakened connectivity in AD patients under specific functional states, accompanied by alterations in state dwell time and transition frequency. Integrative analysis of FG and dFC indicated that the reorganization of brain networks in AD involves both macroscopic gradient alterations at the integration level and reduced flexibility in transitioning across states.

### 4.1 Abnormalities in the principal gradient

The principal gradient represents a functional continuum transitioning from primary sensorimotor regions to higher-order transmodal cortices, serving as a key indicator of the brain’s macroscale functional integration capacity (Huntenburg et al., 2018). We found that abnormalities in AD patients were primarily located along the first gradient, with DMN regions showing reduced gradient scores in the left precuneus and elevated gradient scores in the bilateral medial superior frontal gyrus (mSFG) and angular gyri, while additional alterations also involved the SMN. The precuneus, a core hub of the DMN involved in self-awareness and episodic memory (Cavanna, 2007), showed reduced gradient scores, suggesting diminished functional specificity and decreased segregation from transmodal regions, which may contribute to or reflect cognitive decline in the early stages of AD (Wang Y. et al., 2024). These findings are consistent with previous reports by Zhao et al. (2020), who observed impaired macroscale integration in AD patients. Further analysis revealed that gradient scores in the left precuneus were positively correlated with age, suggesting age-related alterations in the functional organization of this region. While the precuneus showed reduced functional specificity, the right medial superior frontal gyrus exhibited elevated gradient scores, which may reflect a compensatory reorganization aimed at preserving overall network integration. This pattern is consistent with previous reports of compensatory enhancement in the frontoparietal regions (Valera-Bermejo et al., 2020). Notably, we also observed a significant positive correlation between gradient scores in the right medial superior frontal gyrus and T-Tau levels, indicating a potential link between this region’s gradient features and biomarkers of neuronal injury. In addition, its negative correlation with age suggests a degree of sensitivity to age-related decline, which appeared to be more pronounced in patients with AD.

In the angular gyrus, a key transmodal cortical hub, AD patients exhibited elevated gradient scores. The angular gyrus plays a central role in the default mode network (DMN), supporting high-level functions such as semantic processing, scene construction, and self-referential cognition (Seghier, 2023). The observed gradient increases may reflect compensatory reorganization aimed at maintaining network integration and cognitive functions despite underlying disruptions. This finding is consistent with previous reports of compensatory enhancement in frontoparietal regions, suggesting that transmodal cortices in AD may increase their integrative engagement with other regions to partially mitigate cognitive decline. Notably, the right angular gyrus showed a negative correlation between gradient scores and age, indicating that this compensatory reorganization may diminish with advancing age, thereby reflecting reduced plasticity and heightened vulnerability of transmodal cortices in older AD patients.

Building on these findings in the DMN, similar bidirectional alterations were evident within the SMN. Specifically, gradient scores were elevated in the right supplementary motor area (SMA) but reduced in the bilateral superior temporal gyri (STG). This divergent pattern suggests that AD disrupts not only higher-order cognitive hubs but also the macroscale hierarchy underlying sensorimotor control. The increased gradient scores in the SMA may reflect a compensatory reorganization, whereby this region assumes a more transmodal role in motor planning and coordination to offset deficits in other motor-related areas (Glasser et al., 2016). In contrast, reduced gradient scores in the STG likely reflect degeneration of language-action integration circuits, consistent with the decline in verbal fluency commonly reported in AD patients (Makris et al., 2013). Taken together, these abnormalities-predominantly aligned with the first gradient-underscore that functional reorganization in AD extends across multiple networks, destabilizing the perception-cognition-action continuum that supports everyday behavior.

The overall compression of functional gradients observed in Alzheimer’s disease (AD) may reflect large-scale functional dedifferentiation and blurring of hierarchical network organization. In this study, functional gradient alterations in the right medial superior frontal gyrus were significantly and positively correlated with T-Tau levels, suggesting that the disruption of hierarchical organization may be associated with tau-related synaptic dysfunction and reduced coordination of neural activity. This interpretation is consistent with previous neuropathological and neuroimaging findings showing that tau deposition can interfere with synaptic transmission, weaken long-range neuronal coupling (Canuet et al., 2015; Ondrejcak et al., 2023), and consequently impair the hierarchical integration and differentiation of large-scale brain networks. Moreover, the neurotransmitter imbalance commonly observed in AD-characterized by reduced GABAergic inhibition and excessive glutamatergic excitation-may further diminish the brain’s capacity for dynamic functional segregation (Mattson, 2020; Jiménez-Balado and Eich, 2021). Collectively, these neurophysiological disturbances may jointly contribute to cortical gradient compression and decreased segregation of higher-order associative networks, manifesting as large-scale functional dedifferentiation and compensatory hyperactivation in prefrontal regions. These gradient alterations, particularly their association with T-Tau, suggest that hierarchical network metrics may complement established biomarkers in characterizing disease-related network vulnerability, offering a novel perspective on the large-scale functional reorganization observed in this disease.

### 4.2 Abnormalities in the second gradient

In contrast to the widespread abnormalities observed along the first gradient, alterations in the second gradient appeared more regionally restricted. The second gradient is thought to capture cross-modal integration and secondary functional differentiation (Huntenburg et al., 2018), thereby reflecting more fine-grained network specialization. In the present study, abnormalities were confined to the left postcentral gyrus within the SMN and the left middle occipital gyrus within the VIS, both showing reduced gradient scores. The postcentral gyrus, as the primary somatosensory cortex, exhibited diminished gradient values, suggesting impaired hierarchical integration between sensory input and motor feedback. Such alterations may underlie the delayed responses or motor incoordination frequently reported in AD patients (Hartwigsen, 2018). Similarly, the middle occipital gyrus, a higher-order visual association area involved in visual attention and dynamic perception, showed decreased gradient scores, which may be linked to the well-documented spatial navigation deficits and face recognition impairments in AD (Palejwala et al., 2021; Plaza-Rosales et al., 2023). Taken together, the regionally localized abnormalities observed in the second gradient suggest that, beyond the large-scale disruptions of macroscale functional organization, AD also involves more fine-grained hierarchical impairments, which may contribute to the compound nature of its cognitive and perceptual deficits.

### 4.3 Dynamic functional connectivity based on sliding-window analysis

Consistent with the abnormalities revealed by static FG, our dynamic functional connectivity (dFC) analysis further demonstrated bidirectional alterations in AD patients, predominantly in states 2, 3, and 4. In state 2, decreased dFC was observed within parietal-temporal pathways (e.g., inferior parietal lobule-Heschl’s gyrus), suggesting impaired semantic-spatial integration. This finding is in line with previous reports of disrupted parietal-temporal coupling in AD (Makris et al., 2013; Filippi et al., 2019). In contrast, enhanced dFC was found within prefrontal-insula-motor circuits (e.g., orbital superior frontal gyrus-opercular inferior frontal gyrus, precentral gyrus-medial superior frontal gyrus, orbital middle frontal gyrus-postcentral gyrus), which may reflect compensatory recruitment to maintain motor and cognitive control. This compensatory pattern has also been described in prior studies (Weng et al., 2023; Power et al., 2024). In state 3, dFC reductions were identified between the DMN and emotion-related regions (insula-supramarginal gyrus, posterior cingulate cortex-middle temporal gyrus), consistent with previous findings of diminished cross-network coordination (Zhou et al., 2010), and supporting the hypothesis of impaired dynamic interaction between the salience and emotion-processing networks (Krajcovicova et al., 2014). In state 4, more widespread abnormalities were present, including reduced dFC in circuits supporting motor planning (supplementary motor area-caudate), audiovisual integration (superior/middle occipital gyrus-Heschl’s gyrus), and core DMN nodes (posterior cingulate cortex-middle temporal gyrus). These results not only reinforce evidence for DMN reorganization in AD (Zhang et al., 2018), but also extend current knowledge by providing network-level evidence of disrupted sensorimotor integration (Giannì et al., 2021) and impaired multimodal information processing (Unruh et al., 2023).

Furthermore, temporal metrics indicated that AD patients exhibited prolonged mean dwell time (MDT) and increased fractional occupancy (FT) in states 1 and 2, but reduced MDT and FT in state 3, alongside an overall decrease in the number of transitions (NT). This pattern, characterized by longer dwell times and fewer transitions, suggests reduced flexibility in dynamic brain states. Such alterations align with previous reports of impaired state switching and decreased dynamism in AD (Jones et al., 2012; Cabral et al., 2017). The loss of network flexibility may hinder the ability of AD patients to reorganize and switch network states in response to changing cognitive demands, ultimately compromising the execution of complex cognitive functions.

Our findings converge with and extend previous dynamic functional connectivity studies in Alzheimer’s disease, which have consistently reported reduced network flexibility, prolonged dwell time in specific functional states, and fewer transitions across the AD continuum (Jones et al., 2012; Gu et al., 2020; Tessadori et al., 2025). Such temporal alterations are closely associated with cognitive decline and disease stage, underscoring the sensitivity of dynamic network metrics to AD-related dysfunction. By linking state-specific temporal features to network-level functional gradient alterations, the present study introduces a hierarchical spatial perspective, indicating that impaired state switching in AD preferentially affects networks with compressed or shifted gradients, including the default mode, sensorimotor, and visual systems.

### 4.4 Dual patterns of alterations in FG and dFC

When considered together, the FG and dFC results revealed two distinct patterns of alterations in AD patients. In the visual network (VIS), the left middle occipital gyrus showed reduced gradient scores accompanied by decreased dynamic connectivity with the transverse temporal gyrus. This “gradient decrease-connectivity decrease” pattern suggests that diminished hierarchical organization in this region was not compensated by enhanced inter-regional coupling, potentially limiting its capacity for cross-network integration. Such changes are in line with the spatial disorientation and impaired visual recognition frequently reported in AD patients (Sophie et al., 2014). By contrast, in the sensorimotor network (SMN), the left postcentral gyrus exhibited reduced gradient scores but enhanced dynamic connectivity with prefrontal regions, such as the orbital part of the middle frontal gyrus. This “gradient decrease-connectivity increase” pattern may indicate that when local functional specialization is weakened, the brain engages additional cross-regional coordination to maintain basic perceptual-motor processes (Song et al., 2025). This observation is consistent with prior reports of compensatory activity in motor-related areas during the early stages of AD (Su et al., 2017) supporting the notion that some degree of adaptive regulation may coexist with functional decline.

Taken together, these findings highlight that changes in AD are not uniform across brain networks. Instead, they involve both regions where reduced functional differentiation coincides with decreased connectivity, and others where reduced gradients are accompanied by compensatory increases in connectivity. Such heterogeneous patterns underscore the importance of jointly considering hierarchical organization and temporal dynamics when characterizing network reconfiguration in AD, as this dual perspective provides a more comprehensive understanding of the interplay between functional impairment and potential compensatory responses (Wang et al., 2021). Notably, similar dual patterns of altered gradients and dynamic connectivity have also been reported in vascular cognitive impairment, such as patients with asymptomatic carotid stenosis (Wang et al., 2025), further supporting the generalizability of this joint framework across different neurological disorders.

### 4.5 Translational and clinical implications

Beyond their mechanistic significance, the present findings also carry potential translational and clinical relevance. FG and dFC metrics are not intended to replace simple cognitive screening tools for distinguishing patients with AD from healthy controls. Rather, their strength lies in capturing subtle alterations in large-scale network topology and temporal dynamics that may be highly sensitive to disease progression and therapeutic modulation.

Functional gradient features, together with dFC-derived temporal metrics such as state-specific dwell time and network flexibility, may therefore serve as promising imaging biomarkers for monitoring the effects of pharmacological and non-pharmacological interventions targeting synaptic function, neurotransmitter balance, or network excitability (Cabral et al., 2017). Previous studies have demonstrated that dynamic connectivity and network topology measures can detect treatment or disease-related changes that are not yet reflected in conventional cognitive scores or structural imaging, supporting their utility as sensitive outcome measures in clinical trials and longitudinal studies (Pini et al., 2025a).

Moreover, the joint FG-dFC framework may facilitate patient stratification in clinical research. Individuals exhibiting pronounced gradient compression together with reduced dynamic flexibility may represent a subgroup with more advanced network-level involvement, whereas patients with relatively preserved hierarchical organization but emerging dynamic abnormalities may follow a different disease trajectory. Integrating FG-dFC metrics with fluid biomarkers within the ATN framework may thus help define biologically more homogeneous subgroups, improve statistical power for detecting treatment effects, and support precision-medicine approaches in AD (Canal-Garcia et al., 2024).

Finally, the observed association between functional gradient alterations in the medial superior frontal gyrus and T-Tau levels suggests that FG-dFC measures can be anchored to underlying molecular pathology. Although the present study is cross-sectional and based on a modest sample size, these findings support the feasibility of using hierarchical and dynamic network metrics as clinically relevant imaging endpoints that bridge molecular biomarkers, network physiology, and cognitive outcomes. Future longitudinal and interventional studies are warranted to determine whether FG-dFC changes can reliably track therapeutic response and predict disease progression (Pievani et al., 2024).

## 5 Limitations

This study has several limitations. First, the sample size was relatively small. Although demographic characteristics were matched, the limited sample may restrict the generalizability and statistical power of the findings. Future studies should expand the sample size and conduct multi-center research. Second, the cross-sectional design only reflects brain functional states at a single time point and cannot untangle the dynamic evolution of brain networks in AD throughout disease progression. Longitudinal follow-up studies are warranted in the future. Third, potential confounding factors such as medication use, comorbidities, and education level were not fully controlled. These variables may influence functional connectivity and gradient patterns and should be systematically incorporated in subsequent research.

## 6 Conclusion

AD is characterized by macro-scale hierarchical disorganization centered on the principal functional gradient, accompanied by reduced cross-state flexibility and state-dependent connectivity abnormalities. The combined functional gradient-dynamic functional connectivity (FG-dFC) analysis provides complementary spatiotemporal insights and reveals imaging features associated with T-Tau levels and age, offering new perspectives on the neuropathological mechanisms of AD and potential imaging biomarkers. Moreover, these network topology and dynamic connectivity metrics may prove useful for monitoring disease progression, evaluating treatment effects, and stratifying patients in future clinical and interventional studies.

## Data Availability

The raw data supporting the conclusions of this article will be made available by the authors, without undue reservation.

## Footnotes

1
http://www.fil.ion.ucl.ac.uk/spm/

2
http://www.rfmri.org/

3
http://github.com/MICA-MNI/BrainSpace

4
http://www.restfmri.net/forum/DynamicBC
